# Waiting time distribution in public health care: empirics and theory

**DOI:** 10.1186/s13561-015-0061-7

**Published:** 2015-08-25

**Authors:** Sofia Dimakou, Ourania Dimakou, Henrique S. Basso

**Affiliations:** 1 Department of Business Administration, Technological Educational Institute of Athens, Athens, Aigaleo - 12243 Greece; 2 Department of Economics, SOAS, University of London, Russell Square, WC1, London UK; 3 Banco de Espa na, Research Department, Alcalá 48, Madrid, 24014 Spain

**Keywords:** Waiting time, Hospitals, Public health provision, Rationing, Pioritisation

## Abstract

Excessive waiting times for elective surgery have been a long-standing concern in many national healthcare systems in the OECD. How do the hospital admission patterns that generate waiting lists affect different patients? What are the hospitals characteristics that determine waiting times? By developing a model of healthcare provision and analysing empirically the entire waiting time distribution we attempt to shed some light on those issues. We first build a theoretical model that describes the optimal waiting time distribution for capacity constraint hospitals. Secondly, employing duration analysis, we obtain empirical representations of that distribution across hospitals in the UK from 1997–2005. We observe important differences on the ‘scale’ and on the ‘shape’ of admission rates. Scale refers to how quickly patients are treated and shape represents trade-offs across duration-treatment profiles. By fitting the theoretical to the empirical distributions we estimate the main structural parameters of the model and are able to closely identify the main drivers of these empirical differences. We find that the level of resources allocated to elective surgery (budget and physical capacity), which determines how constrained the hospital is, explains differences in scale. Changes in benefits and costs structures of healthcare provision, which relate, respectively, to the desire to prioritise patients by duration and the reduction in costs due to delayed treatment, determine the shape, affecting short and long duration patients differently.

**JEL Classification** I11; I18; H51

## Background

The existence of long waiting lists and high waiting times for elective surgery has been a long-standing issue in many publicly funded healthcare systems in the OECD. As a result, given the public demand for good quality and prompt national healthcare, policymakers have extensively focused on average and excessive waiting time as key performance indicators. Concerns with waiting lists within the public arena motivated health economists to develop different frameworks where waiting lists function as rationing devices (see for instance [1, 2]). Most of this theoretical literature put emphasis on hospitals decisions on optimal expected (average) waiting time, reflecting the benefits of having a single statistic to measure quality and efficiency of public healthcare provision. However, in order to understand and design policies that effectively tackle waiting times, it is essential to explore how the wait and order of patients treated is determined. Consequently, the focus must shift from the mean to the whole distribution of waiting times.

Reflecting the need to increase our understanding of rationing through waiting lists, this paper provides an empirical and theoretical analysis of the entire distribution of patients’ waiting times to investigate how hospitals’ admission patterns for elective surgery affect patients differently and which hospitals’ characteristics drive the duration of treatment.^1^ On the theoretical side, we develop a dynamic supply-side model for healthcare that determines the optimal admission behaviour, its drivers and the overall waiting time distribution. On the empirical side, employing the techniques of duration analysis and Hospital Episode Statistics (HES henceforth) data for 1997/98 – 2005/06 covering the English NHS, we estimate the representations of the whole waiting time distribution of elective patients and take our model to the data to estimate the main hospital structural parameters.

Despite vast empirical variation, we can identify particular admission patterns for elective surgery. First, we observe important differences on the ‘scale’ of admissions. That is, some hospitals manage their lists quicker, having smaller waiting times throughout the whole spectrum of the distribution, than others. Our estimation shows this variation is linked to the degree of capacity constraint of a hospital. Second, we frequently observe large differences on the ‘shape’ of admission rates. There are cases in which hospitals put more effort in treating as many patients as possible quickly (prioritising short waiters), at the expense, however, of a fraction of patients who receive treatment with a significant delay. Other hospitals put more emphasis on medium waiters, whereby patients receive treatment more gradually, but the long right tail of the distribution is eliminated. Our estimation shows that the degree of prioritisation in duration, reflecting a preference of the hospital, is an important driver of the waiting time distribution for short duration patients. The third relevant structural component is the change in costs due to postponed treatment; that is, delaying admissions for elective surgery allows the hospital to manage, plan and allocate its resources more effectively. We find that this is an important factor in explaining the admission patterns of medium duration patients. Hospitals that face a flat cost structure, implying little gain from containing costs by delaying treatment, select to treat a higher proportion of medium duration patients, while hospitals whose costs decay more significantly manage lists by treating a higher proportion of patients of very short and long durations. We finally study an extension to the model that explicitly incorporates less and more severe (complex) cases. That increases the ability of the model to match the observed variability on empirical waiting distributions. By exploring differences in patients’ diagnoses to construct different survival curves we confirm hospitals undergo some degree of clinical prioritisation while admitting patients for treatment, selecting to treat more severe/complex cases first.

Although the literature on healthcare provision and waiting lists and times is vast, empirical and theoretical contributions that look at the overall distribution of waiting times are not common. Particularly, duration analysis is only used in a few number of studies. MacCormick and Parry [3] apply it using data for one hospital in New Zealand and [4] while looking at a subset of hospitals/ operations in Canada. For the UK, [5] use HES data for two years and focus on the UK national waiting targets. Our work expands on the latter in several dimensions. By employing this technique for the UK, using a longer time span, and focusing at the hospital level we identify particular hospital-level patterns of admission as a result of hospital management practices, and importantly link those to a theoretical model of healthcare provision. As such, we are able to estimate healthcare supply-side structural characteristics that drive the empirical waiting time distributions highlighting the trade-off between long and short waiters inherent to the management of waiting lists. Our framework furthers our understanding of hospitals’ admission patterns and may provide valuable insights for successful policy design. In [6] we stress the importance of looking at the entire distribution of waiting times, empirically and theoretically, for a widely used policy designed to reduce waiting times; by analysing the distribution we identify asymmetric effects of waiting time targets that are linked to worse healthcare outcomes.

On the theoretical side the closest analyses to this paper come from [1, 2, 7]. The first two put emphasis on the hospital decisions on optimal expected (average) waiting time, with [2] developing a continuous time dynamic framework. The focal point in [7] lies in the influence of average waiting times on patients’ welfare when prioritisation issues are incorporated. The emphasis of these studies is on average waiting times while we look at the entire waiting distribution. Dixon and Siciliani [8] also look at the entire distribution, mapping the distribution of patients already treated (HES data) with the distribution of patients waiting on the list (waiting list returns). At the steady state, a comparison between the two distributions is performed; however, the waiting time distributions are not derived within a model of hospital behaviour like we do here.

## Theoretical analysis

We investigate elective patients’ waiting time distributions both empirically and theoretically. We first develop a healthcare supply-side model that generates waiting lists as optimal outcomes of capacity constrained hospitals. We obtain a set of theoretical predictions linking the hospital’s key characteristics, namely, the degree of capacity constraint, the preference for patient prioritisation due to duration and the cost structure across durations, to the resulting waiting time distributions.

### Theoretical model

We model a hospital that provides healthcare treatment to maximise the benefits from treatment (utility), subject to a budget constraint and the inflow of patients. Although the theoretical literature on waiting lists in healthcare is sizable, guiding most of our modeling assumptions detailed below, no theoretical framework in the literature focuses on the determination of the optimal distribution of waiting times in its entirety as we do. The theoretical model consists of two main parts: a set of patients that are currently waiting to be treated and a hospital that is the healthcare supplier.

#### Patients

Patients currently in the waiting list, *L*_*t*_, are characterised by the severity of their disease, *s*=1,2,…,*p* and the time they have been on the list, their duration *d*=1,2,…*q*. *s* is increasing in severity and *d* denotes the period elapsed between joining the waiting list of a specialist and admittance for surgery at the hospital. The minimum possible waiting time is one period (*d*=1) and the maximum time is bounded by *q* (patients do not wait indefinitely). At each time *t* hospitals treat *k*_*d*,*s*,*t*_ patients that have been in the list with duration *d* and severity level *s*. Thus, total patients treated at time *t* is given by $k_{t}= \sum _{d} \sum _{s} k_{d,s,t} \in L_{t}$. Also denote $k_{d,t}= \sum _{s} k_{d,s,t}$ as the patients of all severities with duration *d* treated at time *t* and $k_{s,t}=\sum _{d} k_{d,s,t}$ as the patients of severity *s* treated at time *t* across all durations.

We do not explicitly model the demand for healthcare, considering a reduced form relationship where the inflow of patients to the hospital is decreasing in expected duration. The higher the expected waiting time at the beginning of *t* is, the lower the demand for public healthcare.^2^ Formally, the inflow of patients in the list, and equivalently, the demand for elective healthcare at the beginning of time *t* is given by

$$x_{t} = Z - \theta E_{t-1}(d) $$

where *E*_*t*−1_(*d*) denotes the duration patients entering in the list at time *t* expect at time *t*−1 (defined below), and *Z* is the potential demand for healthcare, being a function of a vector of exogenous demand factors. This may include socio-economic conditions and morbidity rates. We assume a proportion *δ*_*s*_ of *x*_*t*_ is the inflow of severity *s*=1,2,…,*p*, with $\sum _{s=1}^{p} \delta _{s} = 1$. Thus *x*_*s*,*t*_=*δ*_*s*_ *x*_*t*_. Finally, the sensitivity of demand for healthcare to expected duration is captured by *θ*.

Before we describe the hospital’s main features we briefly present the key theoretical representations of the patients’ waiting time distribution. Here, waiting time is modeled as a discrete variable, where a period of time is equivalent to a month. The probability function of waiting time depicts the whole spectrum of the relative frequencies of patients of severity *s* having waited distinct periods of time until treatment at *t*, *f*(*d*∣*s*)=*P*(*D*=*d*∣*s*). The cumulative function corresponds to the probability of having waited *d* periods or less, *F*(*d*∣*s*)=*P*(*D*≤*d*∣*s*). From here we obtain the two main representations of waiting time distributions used in our study, namely the survival and hazard functions. The survival function shows the probability of a person remaining (surviving) on the list beyond a given time and is indicative of cumulative rates of treatment. We derive the survival function as the complement of the cumulative function, that is *S*(*d*∣*s*)=1−*P*(*D*≤*d*∣*s*)=*P*(*D*>*d*∣*s*). The hazard function is the risk of ‘failure’ at some time *t*. Essentially, it shows the rate at which patients leave the waiting list at a given time, conditional on having waited in the list up to that point. It thus approximates the conditional instantaneous probability of admission, rather than the unconditional one (*f*(*d*∣*s*)). Thus, *h*(*d*∣*s*)=*P*(*D*=*d*|*D*≥*d*,*s*). Table 1 shows the different formats of the waiting time distribution.

**Table 1:** Theoretical waiting time distribution for severity *s*

d	*f*(*d*∣*s*)	*F*(*d*∣*s*)	Survival Function	Hazard Function
	*P*(*D*=*d*∣*s*)	*P*(*D* ≤ *d*]∣ *s*)	*P*(*D* > *d* ∣ *s*)	*P*(*D*=*d*|*D* ≥ *d*,*s*)
0	0	0	1	0
1	$\frac {k_{1,s,t}}{k_{s,t}}$	$\frac {k_{1,s,t}}{k_{s,t}}$	$1-\frac {k_{1,s,t}}{k_{s,t}} \,=\, \frac {\sum _{d=2}^{q}k_{d,s,t}}{k_{s,t}}$	$\frac {k_{1,s,t}}{\ k_{s,t}}$
2	$\frac {k_{2,s,t}}{k_{s,t}}$	$\frac {k_{1,s,t}+k_{2,s,t}}{k_{s,t}}$	$1-\frac {k_{1,s,t}+k_{2,s,t}}{k_{s,t}} \,=\, \frac {\sum _{d=3}^{q}k_{d,s,t}}{k_{s,t}}$	$\frac {k_{2,s,t}}{\sum _{d=2}^{q}k_{d,s,t}}$
·	·	·	·	·
·	·	·	·	·
*q*−1	$\frac {k_{q-1,s,t}}{k_{s,t}}$	$\frac {\sum _{d=1}^{q-1} k_{d,s,t}}{k_{s,t}}$	$\frac {k_{q,s,t}}{k_{s,t}}$	$\frac {k_{(q-1),s,t}}{k_{(q-1),s,t}+k_{q,s,t}}$
*q*	$\frac {k_{q,s,t}}{k_{s,t}}$	1	0	1

For simplicity we assume potential patients do not know their severity.^3^ Thus, in order to obtain the expected duration they look at all patients treated. As such expected waiting time at time *t* under rational expectations is given by

$${} {\fontsize{8.8pt}{9.6pt}\selectfont{\begin{aligned} E_{t-1}^{RE}(d) &= E_{t-1} \left(\sum_{d=1}^{q} d \, \frac{k_{d,t+d-1}}{x_{t}} \right)\\&= E_{t-1} \left(\!1 \times \frac{k_{1,t}}{x_{t}} + 2 \times \frac{k_{2,t+1}}{x_{t}} + \ldots + q \times \frac{k_{q, t+(q-1)}}{x_{t}}\!\right). \end{aligned}}} $$

#### Hospital

The two key features of the hospital in our model are the benefits of providing treatment (utility) and its cost structure.

#### The utility of the hospital

The hospital’s utility from healthcare provision, or the benefits from treatment of an altruistic hospital, at any point in time *t*, is given by

(1) $$  U_{t} = g(k_{t}) = \sum_{d} \sum_{s} g(k_{d,s,t}).  $$

*g*(*k*_*d*,*s*,*t*_) denotes the hospital’s (monetary or non-monetary) gain from treating *k* patients of severity *s* and duration *d*. Recall that here the waiting time (*d*) is not a choice variable, but is endogenously determined. The hospital chooses optimally the number of patients of each severity and duration to be treated at time *t*, and this choice determines the waiting time implicitly. We make three general assumptions on the sensitivity of the hospital’s utility to treatments of different severity and durations.

##### Assumption 1.

For a given number of patients treated of the same severity level (i.e. fixed *k* and *s*), the higher the waiting time, the lower the hospital’s utility. That is,

$${} \frac{\partial g(k_{d, s, t})}{\partial d}<0 \quad \text{or} \quad g(k_{d_{1},s,t}) > g(k_{d_{2},s,t}) \quad \text{for \(d_{2}>d_{1}\)}. $$

##### Assumption 2.

For a given number of patients treated of the same duration (i.e. fixed *k* and *d*), the higher the patient’s severity, the higher the hospital’s utility. That is,

$$\frac{\partial g(k_{d, s, t})}{\partial s}>0 \quad \text{or} \quad g(k_{d,s_{1},t}) < g(k_{d,s_{2},t}) \quad \text{for \(s_{2}>s_{1}\)}. $$

##### Assumption 3.

For the same *d* and *s*, *g*(*k*_*d*,*s*,*t*_) is concave in *k*_*d*,*s*,*t*_∈[0,*k*] and exhibits a turning point.

The main rationale for each assumption respectively is: (*i*) hospitals prefer to treat as many people as possible sooner rather than later since later treatments generate less benefits to patients (see for instance [1] and [2]); (*ii*) hospitals are willing to prioritise by the degree of severity, selecting treatment based on clinical need according to NHS core principles; (*iii*) hospitals prefer to spread treatment across different durations implicitly recognising that this allows for a better management of capacity and resource utilisation, increasing the hospital’s gains from treatment (see [1, 2]^4^).

#### The cost of the hospital

With respect to the cost of healthcare provision, we assume that the hospital is capacity constrained and has a budget allocated for elective surgeries given by *B*_*t*_. The hospital’s cost from providing healthcare can be decomposed into two separable parts.

(2) $$  C_{t} = c(k_{t}; \bar{k}) + \sum_{d}\sum_{s} c(k_{d,s, t}).  $$

The first part, $c(k_{t}; \bar {k})$, is the hospital’s scale cost, while the second its duration and severity specific cost, denoted by *c*(*k*_*d*,*s*,*t*_). A similar separation between non-surgical and surgical (directly related to treatment and thus severity and duration) is done by [9]. $c(k_{t}; \bar {k})$ is a function of the overall number of treated patients (*k*_*t*_) in relation to the number of patients ($\bar {k}$) the hospital can treat given its physical capacity or capital (we will generally call $\bar {k}$ the hospital’s capacity). When the potential demand for health (*Z*) is greater than capacity ($\bar {k}$), the hospital cannot treat all the new patients that demand elective healthcare at *t*, being capacity constrained, and thus a waiting list and waiting times emerge.^5^ In addition, whenever optimal $k_{t}>\bar {k}$, the hospital operates above its capacity, increasing the utilisation of its resources.

We make three key assumptions on the cost structure of hospitals. Assumption 4 relates to hospital’s scale cost and Assumptions 5 and 6 to hospital’s patient-specific cost.

##### Assumption 4.

Once the capacity limit of the hospital is reached, the scale cost, $c(k_{t}; \bar {k}, \tau)$, is increasing in *k*_*t*_.

##### Assumption 5.

For the same severity and a given number of treated patients, treating quicker is more costly. That is,

$$\frac{\partial c(k_{d, s, t})}{\partial d}<0. $$

##### Assumption 6.

For the same waiting time and a given number of treated patients, treating more severe cases is more costly. That is

$$\frac{\partial c(k_{d, s, t})}{\partial s}>0. $$

The main rationale for each of these assumptions respectively is: (*i*) treating more patients relative to the limit imposed by the hospital’s physical capacity becomes increasingly costly; (*ii*) costs decrease monotonically with duration^6^; (*iii*) for the same duration and number of treatments, hospital’s cost is increasing in patients’severity (see [7, 10, 11] for frameworks that also incorporate severity levels).

### Hospital’s maximisation problem

In order to facilitate notation of the hospital’s problem, let the number of patients of duration *d*>1 and severity *s* currently waiting for treatment at time *t* be *Ψ*_*d*,*s*,*t*−1_. This stock is equal to the inflow of patients in time *t*−*d*+1 minus all patients treated during periods *t*−*d*+1 until *t*−1. Formally, we define^7^

$$\Psi_{d,s,t-1} = x_{s,t-d+1} - \sum_{j = 1}^{d-1} k_{d-j, s,t-j}. $$

The hospital maximises its utility function, *g*(*k*_*d*,*s*,*t*_), selecting *k*_*d*,*s*,*t*_ for all *d* and *s* at time *t* subject to its constraints,

$$\begin{array}{*{20}l} \max_{\{k_{d,s,t}\}_{d,s}}& \, E_{0}\sum_{t=0}^{\infty} \sum_{d=1}^{q} \sum_{s=1}^{p} g(k_{d,s,t}) \\ \text{Subject to} \quad & \sum_{d} \sum_{s} c(k_{d, s, t}) + c(k_{t}, \bar{k}) \leq B_{t}\\ & 0 \leq k_{d,s,t} \leq \Psi_{d,s,t-1}\\ & x_{t} = Z-\theta E_{t-1}(d) \\ & \Psi_{d,s,t} = 0\; \text{for}\; d > q \end{array} $$

The first constraint corresponds to the budget constraint of the hospital. Here and unlike in [12] the budget allocated to the hospital is exogenously given^8^ and thus our basic set-up is closely linked to the non-cooperative game of [1]. The second constraint states that the amount of patients of duration *d* and severity *s* treated at time *t* (*k*_*d*,*s*,*t*_) must be between zero and the number of untreated patients in the list for that duration and severity. In other words, the number of people selected for treatment at time *t* cannot exceed the corresponding number of people waiting. Third, the hospital takes the evolution of patients inflow into account, and lastly we impose that the maximum waiting time is *q*. We solve this problem assuming a steady state has been reached (see the Appendix for details) and thus the number of entries to the list is equal to the number of patients treated at any point in time (*x*_*t*_=*k*_*t*_) and the optimal *k*_*d*,*s*,*t*_ are time-invariant. At the steady state the expected waiting time becomes

$${} \begin{aligned} E_{t-1}(d) =\bar{d} = \sum_{d=1}^{q} d\ f(d) = \sum_{d=1}^{q} d \, \frac{k_{d}}{k} &= 1 \times \frac{k_{1}}{k} + 2 \times \frac{k_{2}}{k}\\ &\quad+ \ldots + q \times \frac{k_{q}}{k}. \end{aligned} $$

### Functional forms

Following the restrictions implied by Assumptions 1-6, generally accepted by the literature, we start by assuming a set of functional forms and parameters for the key elements of the model and draw conclusions on the link between hospital characteristics and their respective waiting time distributions. In the empirical section we estimate a subset of those parameters, comparing hospital’s cost, benefits and capacity constraints across the English NHS.

The utility of the hospital, $U = \sum _{d} \sum _{s} g(k_{d, s})$ is a function of (*d*×*s*) variables and the main specification for *g*(*k*_*d*,*s*_) is assumed to be a third order polynomial,

$$g(k_{d,s}) = a_{d,s}k_{d,s}^{3} + b_{d,s}k_{d,s}^{2} + c_{d,s}k_{d,s}, $$

where *a*_*d*,*s*_<0, *b*_*d*,*s*_>0, *c*_*d*,*s*_>0 are functions of duration and severity such that $\frac {\partial a_{d,s}}{\partial d} >0$, $\frac {\partial b_{d,s}}{\partial d} <0$, $\frac {\partial c_{d,s}}{\partial d} <0$ and $a_{d,s_{2}}\geqslant a_{d,s_{1}}$, $b_{d,s_{2}}\leqslant b_{d,s_{1}}$ and $c_{d,s_{2}}\leqslant c_{d,s_{1}}$ for *s*_2_>*s*_1_, with at least one with strict inequality. This specification fulfills Assumptions 1-3 laid out above. In the following subsection, we will allow for two extra functional forms of the utility function of the hospital *g*(*k*_*d*,*s*,*t*_) and analyse their implications: (a) a monotonically increasing function with increasing rates (quadratic) and (b) a monotonically increasing function with decreasing rates (logarithmic). In both of these cases assumption 3 is relaxed. On the cost side, the hospital is faced with a scale cost, as well as a cost specific to the duration and severity of each treatment. Both specifications below conform with assumptions 4-6. The scale cost is given by^9^

$$c(k) = \tau(k-\bar{k})^{2}. $$

*τ* reflects the cost sensitivity of operating above physical capacity. This parameter can be interpreted as the efficiency of the hospital in operating its physical capacity (allocating the number of beds to patients or the surgery theaters to procedures efficiently), by treating more or less patients without utilising as much of the budget assigned by the NHS.^10^ The default duration and severity specific cost is linear on numbers of patients treated and given by

$${} \begin{aligned} &c(k_{d, s}) = \rho_{d,s} k_{d,s}, \quad \text{with }\frac{\partial \rho_{d,s}}{\partial d}\left<0 \text{and} \frac{\partial \rho_{d,s}}{\partial s}\right>0, \text{where} \rho_{d,s}\\ &\qquad\;\;= \frac{{\rho^{0}_{s}}}{d^{{\rho^{1}_{s}}}},\text{such that} \\ &{\rho^{0}_{s}} \text{controls the scale and} {\rho^{1}_{s}} \text{the sensitivity of cost to}\\& \text{duration or duration decay.} \end{aligned} $$

### Theoretical implications

#### Results - benchmark

Our benchmark framework employs the simplest case in which patients are not differentiated by the severity of their condition. The default parameterisation is depicted in Table 5 in the Appendix. For simplicity we set *q*=36 periods (maximum duration - 36 months). The solution to the hospital’s problem and the corresponding waiting time distribution is obtained numerically.^11^

**Fig. 1:**
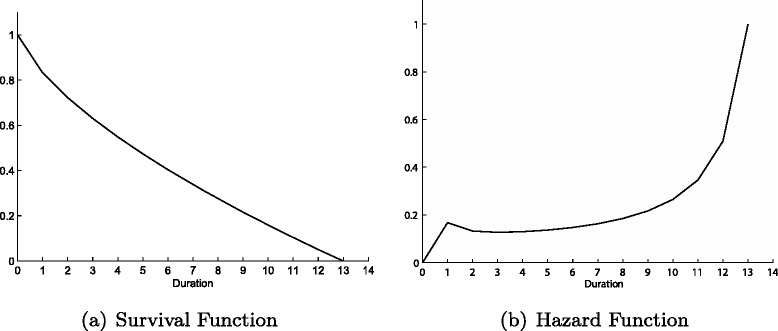
Survival (**a**) and Hazard (**b**) functions for the Benchmark Model

The main output of the model are two representations of the waiting time distribution: the survival curve (a) and the hazard curve (b) depicted in Fig. 1. The survival curve starts from one, as all patients are waiting to be treated at duration zero, and then decreases monotonically while the hospital removes patients off the list, reaching zero at *d*=13. The hazard curve exhibits a spike at *d*=1, and after waiting period two, it increases monotonically until one. The observed decline between durations one and two is due to the largest proportion (0.168) of treatments taking place in period one.

The mechanisms that drive such an admission pattern depend mainly on the interactions of hospital’s costs, utility and patient inflow. The hospital would prefer to treat as many patients as possible immediately, however this comes at a higher cost. Additionally, given the cubic specification assumed, the turning point in each utility curve for *d*=1,2,…,*q* serves as a natural threshold for the amount of patients selected from each duration. In particular, this feature restrains the hospital from excessively ‘front-loading’ treatments. The third factor that restricts the hospital from treating too many patients up front is the impact of a small expected waiting time on future inflow. If the list is cleared quickly, expected duration will be low and a higher number of patients will demand healthcare in the following period. As the hospital is capacity constrained, that would lead to increasing waiting lists such that in the future it might be unable to continue treating patients of short durations or the list may get explosive. Therefore, the hospital may delay treatment today to avoid too high inflow relative to capacity in the future. In order to clarify these forces underlying the hospital’s behaviour we solve the model for different parameters and functional forms, highlighting the importance of capacity constraints, prioritisation and sensitivity of costs due to duration.

#### Capacity constraint

**Fig. 2:**
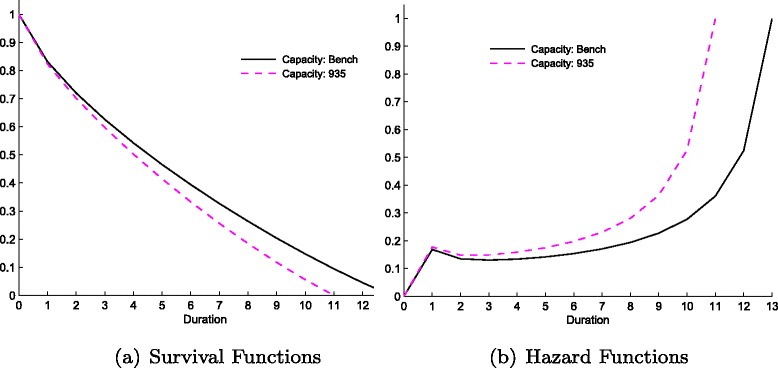
Survival (**a**) and Hazard (**b**) functions from changes in the hospital’s capacity

In this specification we measure the effects of increasing the hospital’s capacity, as measured by the maximum number of patients, $\bar {k}$, holding the inflow of patients *Z* constant. Define the ratio of $Z/\bar {k}$ as the degree of capacity restriction of a hospital. With higher capacity, the hospital has the ability both to treat more patients and to treat them faster. As the degree of capacity restriction reduces from 33 % to 28 %, the total number of patients treated increases by around 4 % and at the same time the expected waiting time decreases from 5.66 to 4.98 months. Increased capacity produces a clear scale effect; more patients with short waits are admitted for treatment, while long waiters (*d*=12,13) are eliminated. The survival curve shifts towards the origin (see Fig. 2(a)), as admission rates rise throughout. Regarding instantaneous rates, together with the ‘usual’ hump at the beginning, the hazard curve shifts leftwards (Fig. 2(b)).^12^ We find similar scale effects when we increase, ceteris paribus, the budget (*B*).^13^ Thus, the distance of survival curves from the origin are related to the degree of capacity constraint hospitals face measured by the ratio of $Z/\bar {k}$.

#### Patient prioritisation

**Fig. 3:**
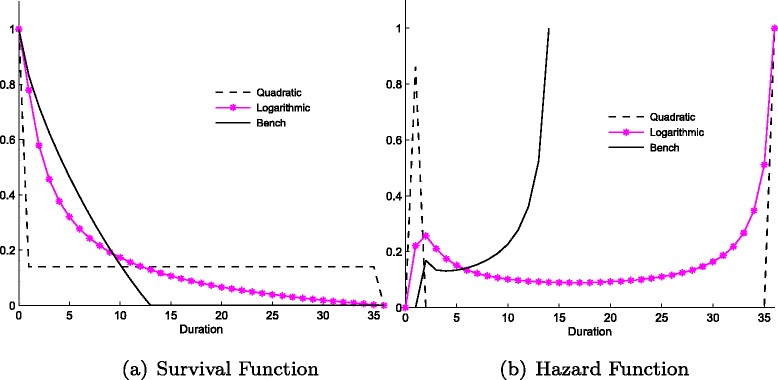
Survival (**a**) and Hazard (**b**) functions for quadratic and logarithmic utility specifications

The utility of the hospital determines the overall preference for treating patients at different points in time. In order to verify the relationship between benefits and survival curves we change the form of the utility function, relaxing Assumption 3. Instead of the third order polynomial used in the benchmark, we employ a quadratic function, $U(k_{d}) = b_{d} {k_{d}^{2}} + c_{d}k_{d} + e$, thus setting *a*_*d*_=0 for all *d*, and a logarithmic function, *U*(*k*_*d*_)=*γ*_*d*_ *l* *o* *g*(*k*_*d*_+1). Results are presented in Fig. 3.

With a quadratic utility function, the majority of patients are treated within the same period, while the rest receive treatment at the largest possible duration. Intuitively, since the quadratic utility curve has no turning point and since $\frac {\partial u(k_{1})}{\partial \hat {k}}>\frac {\partial u(k_{n})}{\partial \hat {k}}$ for ∀ *n*>1, the hospital treats as many patients as possible with duration one (given the costs and the capacity it faces). However, the remaining patients are treated at the maximum possible waiting time, since this is the only way to maintain a steady state average waiting time and inflow, and minimise costs. Consequently, the survival graph becomes a one-step function, since, in our example, 86 % of patients are treated with duration one, and the rest after having waited for 36 periods. In sharp contrast, a logarithmic utility function delivers a very smooth steady state waiting time distribution, in which the hospital treats patients in each duration. Again, the number of treated patients is decreasing in *d*, with more treated up front, however, as the logarithmic utility curves are increasing at a decreasing rate (with no turning point compared to the benchmark), utility is maximised when a decreasing number of patients, *k*_*d*_, is admitted from each *d*.

These two functional forms serve as the two extremes of the hospital behaviour as regard prioritisation (treating as many patients as possible with low duration), highlighting the trade-off in place. On the one hand, hospitals have an incentive to ‘front-load’, treat as many patients as possible in the first few periods. On the other hand they must ensure they can deal with the current inflow (without an ever-increasing waiting list) and monitor costs. Therefore, if the first incentive is strong enough (quadratic), survival functions become a step-function. Otherwise when utility gains do not change as dramatically with duration, survival functions are very smooth (logarithmic). The intermediary case occurs with the third order polynomial, whereby front loading is optimal but utility from treating too many patients quickly is low, forcing medium duration patients to be treated as well. Thus, the lower ∣*a*_*d*_∣, the term controlling the third order term of the polynomial, the higher is the prioritisation hospitals are willing to do, although waiting lists as a result become longer, increasing the convexity of survival curves.

#### Duration sensitivity of costs

In this specification we alter the duration-specific cost of the hospital by allowing the unit cost of treatment for each duration to increase, while its budget remains the same.^14^ The unit cost of treatment for *d*=1 is always the same and equal to 20. In the alternative scenario, as *d* increases, the cost of treatment of longer waiters is declining slowly, which implies a higher cost for treating patients with short, as well as medium durations, relative to the benchmark case (see Table 2). We call the alternative scenario *flat* since it represents a flatter unit cost function comparing to the benchmark case.

**Table 2:** Changes in *ρ*_*d*_: Cost of one treatment for the first ten months

	*ρ*_*d*_∖*d*	1	2	3	4	5	6	7	8	9	10
*Benchmark*	$\frac {20}{d^{2}}$	20	5	2.22	1.25	0.80	0.56	0.41	0.31	0.25	0.20
*Flat*	$\frac {20}{d^{0.6}}$	20	13.20	10.35	8.71	7.61	6.83	6.22	5.74	5.35	5.02

**Fig. 4:**
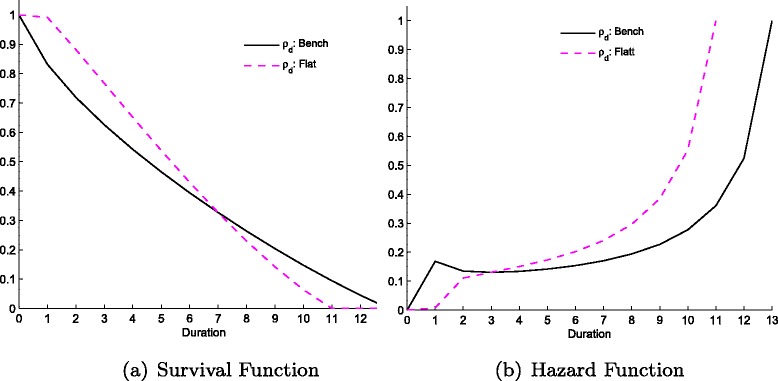
Survival (**a**) and Hazard (**b**) functions from changes in *ρ*_*d*_

As shown in Fig. 4, under the flatter curve it is relatively more costly to treat a significant amount of patients in the first few periods of wait. As a result, the hospital starts by treating only a few patients within the first periods (low decay in the survival curve), treating 14 % less patients in the first 3 periods compared to the benchmark case. After that, the decreasing feature of the cost structure induces the hospital to adopt the ‘typical’ admittance pattern in which the number of treatments decreases with duration. Decreasing the sensitivity of costs to duration (duration decay) holding that initial costs of treating patients constant (*ρ*_0,*d*_) exacerbates the trade-off between shorter and longer duration patients, producing concave survival curves for low *d*.

### Extension: incorporating severity levels

**Fig. 5:**
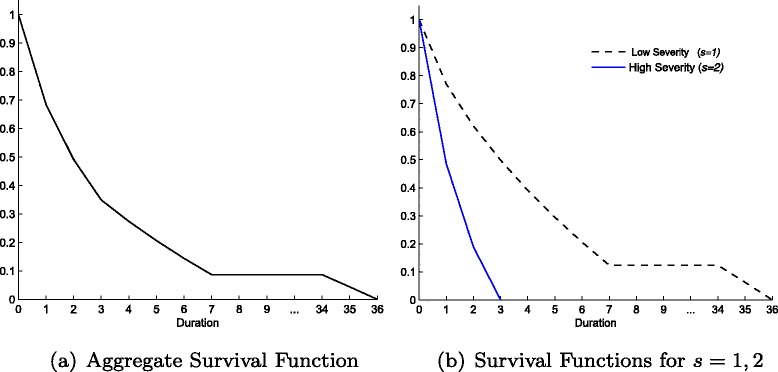
Aggregate (**a**) and for each severity (**b**) Survival functions with two severity levels (Table 3)

Our analysis is extended to incorporate different severity levels. Patients are differentiated according to the level of severity of their health condition. For simplicity we consider two types of severity (*s*=1, low) and (*s*=2, high). This allows for some degree of clinical prioritisation of the list as in [7]. The parameterisation for the new model is given in the Appendix, and Table 3 and Fig. 5 below present the steady state optimal waiting time distribution under the benchmark.

**Table 3:** Optimal steady state results with two severities: *s*=1, low and *s*=2, high

Duration	Optimal *k*_*d*,1_	Optimal *k*_*d*,2_	Agg. *k*_*d*,*s*_
0	0	0	0
1	147.922	141.765	289.686
2	96.075	81.802	177.877
3	78.818	52.199	131.016
4	68.982	-	68.982
5	62.223	-	62.223
6	57.045	-	57.045
7	52.847	-	52.847
8	-	-	-
⋮	-	-	-
35	39.219	-	39.219
36	40.322	-	40.322
*k* ^∗^	643.45	275.765	919.218
*E*(*d*)	7.3044	1.6752	5.6156

The more severe cases have a higher utility gain, but at the same time are more costly (for any given *d*). Given the magnitude of those two trading-off forces the hospital admits for surgery the severe cases (30 % of the overall treatments) much quicker (*q* ^∗^=3 and average duration at 1.6). At the same time the hospital treats less severe cases in a pattern similar to the benchmark but exhibits a long right tail. The overall number of treatments is 919 and the overall average waiting time is 5.6 periods, although milder patients wait on average much more than the ones facing a more serious condition (Table 3). Thus, the hospital prioritises the more severe cases. However, given the resources/budget available and the higher cost for the quicker treatment of the more severe patients (*c*(*k*_*d*,2_)), some of the milder cases are prolonged until the maximum possible duration.

As shown in Fig. 5, the survival curve for the more severe patients is very close to the origin, decreasing quite steeply and reaching zero after only three periods of wait. On the other hand, the survival function for the milder cases is further away from the origin throughout, decreasing much slower until *d*=7, after which point it flattens until the last 80 patients in the list are treated. The aggregate survival curve still displays the same long right tail, however, we also observe a change in the rate of decrease with admittance rates relatively larger for the first three durations, slowing down after that.

**Fig. 6:**
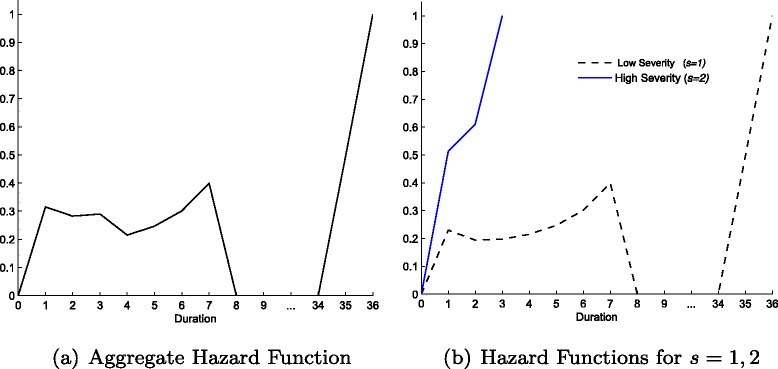
Aggregate (**a**) and for each severity (**b**) Hazard functions with two severity levels (Table 3)

Figure 6 shows the hazard functions. The majority of the treatments take place within the same period (*d*=1), thus, the aggregate hazard curve decreases between *d*=1 and 2. In addition, since the more severe cases are treated within the first three periods, we observe a second drop in the hazard function between durations 3 and 4. After that the conditional probability of being treated keeps increasing until duration 7, drops to zero for the next 28 periods and reaches one at duration 36. The framework produces more changes in slopes of survival functions and more volatile hazard functions as the ones depicted in the simple benchmark, thus the introduction of different severity levels and clinical prioritisation of care increases the flexibility of the model in matching the empirical waiting time distributions observed in the NHS, discussed in the following section.

## Empirical results and discussion

Building upon the main theoretical insights of our model, we now analyse empirically hospital-level waiting time distributions and link those to hospital characteristics estimating the parameters of our model that control the degree of capacity constraint, the degree of prioritisation and finally the cost structure. Before we discuss the main empirical results we briefly describe our dataset.

### Data

The HES is the database employed. This covers all NHS hospital patients treated in a given financial year in England and Wales, recording both the date the patient was placed on the waiting list and the treatment date. The difference between the two serves as the measure of waiting time (or duration). HES data also provide additional information on specialty, diagnosis, operation, type of admission (waiting list, booked and planned) and length of stay. We evaluate three specialties (general surgery, trauma and orthopaedics, and ophthalmology) consisting of more than 50 % of patients waiting for elective surgery. The time coverage is nine years from 1997/98 until 2005/06. We use this data set for two empirical exercises. The first, employing duration analysis, estimates waiting time distributions. The second uses the latter empirical distributions and a minimum distance method to estimate the parameters of our theoretical model, obtaining a measure for the key hospital’s characteristics that drive treatment plans.

### Empirical waiting times distributions: exploring shape and scale

For the first empirical exercise we employ duration (also known as time-to-event or survival) analysis to obtain empirical representations of patients’ waiting time patterns.^15^ Duration analysis, by exploring conditional probabilities of treatment and the cumulative density function, is a robust and informative approach, allowing for an in-depth exploration and comparison of distinct admission behaviours. The two key representations of interest, following our theoretical model closely, are the survival and hazard functions. The survival function is estimated using the non-parametric Kaplan-Meier (KM) or product limit estimator [13], while an estimate of the hazard function is obtained as a weighted Kernel density. Comparisons are then performed using both graphical techniques and log-rank statistical tests to ensure the survival curves obtained are statistically different.

Since the aim of the analysis is to examine the variability of waiting time distributions across hospitals, data are classified according to size and type of NHS trust. Taking under consideration information on NHS trust clustering by the Department of Health we classify hospitals by size (large, medium and small acute) and type (acute, specialist and teaching). The same key admission patterns are identified for all classifications, consequently, we only present below the results from a selection of them. Additional and qualitatively similar results are shown in the Appendix.^16^

#### Large acute hospitals

**Fig. 7:**
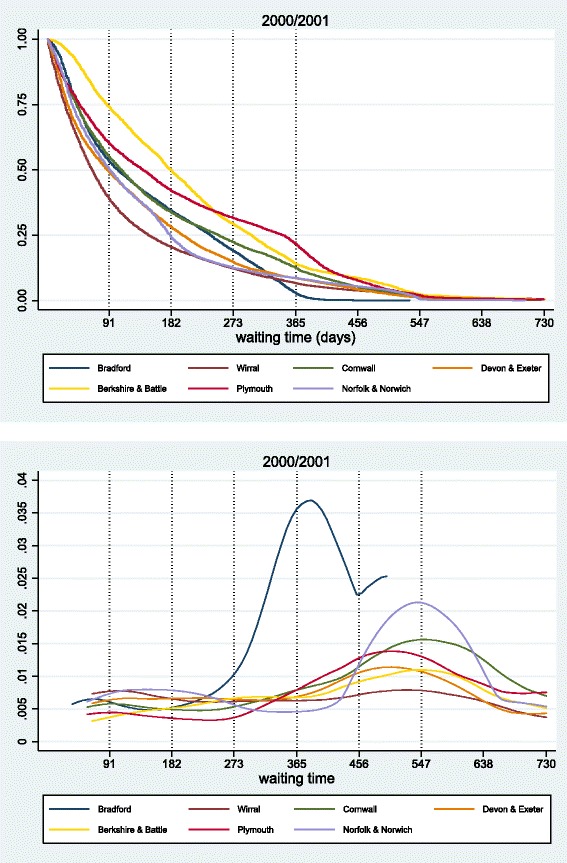
Survival *(top)* and Hazard *(bottom)* curves for large acute hospitals, 2000/2001

We first present the survival and hazard curves for seven large acute trusts for year 2000/01 (Fig. 7). Although we observe curves with different patterns for each hospital in all cases at about 600 days of wait, the proportion of patients on the list has approached zero.

Firstly, we look at scale differences. There are hospitals with higher admission rates throughout the period, thus their survival curve is always closer to the origin. Norfolk & Norwich lies to the left of Berkshire & Battle; after 182 days of wait, there are still 50 % of patients waiting to be treated in the latter, while only 25 % in the former. Secondly, we observe differences in the shape. There are cases where survival curves intersect, indicating a reversal in treatment rates. For example, although Devon & Exeter is admitting patients quicker than Bradford up until 325 days, after that Bradford hospital treats patients with long waits faster. Furthermore, while Wirral’s survival curve is decreasing smoothly, also reflected in a somewhat constant hazard rate, the survival curves for Bradford, Norfolk & Norwich or Plymouth exhibit considerable variation in their slope (size and sign of the second derivative) which translate into more volatile hazard curves.^17^

In terms of hazard curves, we sometimes see a mild hump at short times of wait, and a more distinctive one at longer durations. The first hump, that indicates intensive admission rates at very short waiting times, is suggestive of some form of prioritisation of more urgent cases (see the Appendix for other cases of visible first period humps). For all the large acute hospitals (apart from Bradford), the later peak occurs in the proximity of 547 days, which coincides with the maximum NHS waiting time target for 2000/01 (18 months).^18^ Note that while the theoretical hazard curves approach unity at large durations (since the waiting list is cleared at the steady state), this is not the case in practice as some long waiters are still waiting to be treated. Other than that, however, the theoretical and empirical hazard and survival curves are qualitatively matched.

#### Orthopaedic hospitals

**Fig. 8:**
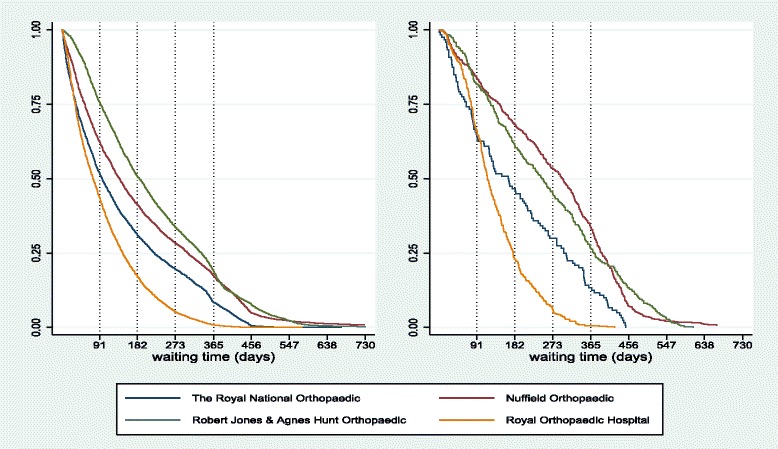
Survival curves hospital level-*left graphs* and hip replacements-*right graphs* in four orthopaedic hospitals for 2002/2003

We now turn to specialist hospitals. We report the survival curves for all procedures for four orthopaedic hospitals (left panel of Fig. 8) but also the waiting distribution for a specific procedure, total hip replacements (right panel of Fig. 8). Survival curves of specialist trusts follow similar patterns as for acute hospitals. Some treat patients quickly for all durations (Royal Orthopaedic Hospital), displaying a convex survival curve and others concentrate treatment to medium durations, selecting not to treat patients of short durations to better manage lists (Robert Jones & Agnes Hunt Orthopaedic), thus having a concave survival curve for short durations. More importantly, we observe that the differences among hospitals persist even when we control for the same treatment procedure (which presumably implies similar resource requirements across hospitals). Hence, the results indicate that the differences in the order of treatment are more likely linked to variations in hospital characteristics.

#### Differentiation by patients diagnoses

The extension to our theoretical model allows for differentiation by patient’s severity, showing how hospitals manage their lists when having to treat both milder cases, and more severe ones that require more attention and resources. In Section ‘Extension: incorporating severity levels’, we saw that the hospital attempts to treat the more severe cases faster, and as a consequence it may delay treatments of milder cases. Theoretical survival and hazard curves become richer, with the latter exhibiting a wider hump at short lengths of wait (see Fig. 6). We attempt to utilise patient-level information from our HES data in order to draw some insights on the actual hospitals’ admission pattern based on complexity of cases.

Using information available on Complications and Comorbidities (CC3)^19^ we provide results that differentiate patients by the complexity of their diagnoses. We first classify HES episodes into the ones identified as exhibiting major complications, looking up until the sixth secondary diagnosis of each patient, and then categorise patients into four groups: the ones that had no complications and the ones with a small (1 or 2), medium (3 or 4), and large (5 and above) number of CC3 indicators.

**Fig. 9:**
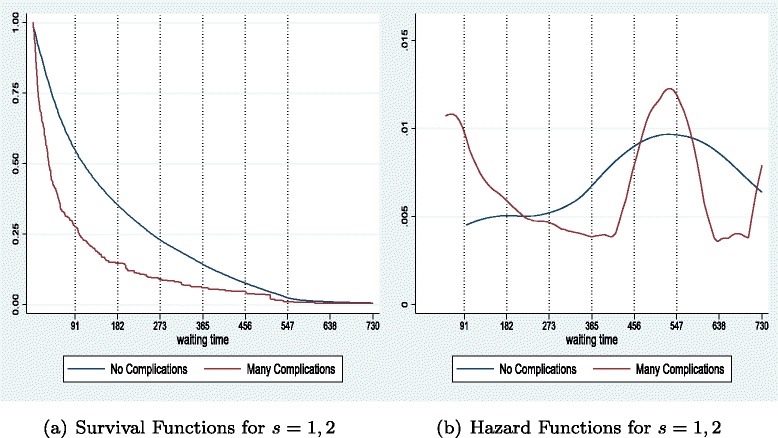
Survival (**a**) and Hazard (**b**) curves by number of complications for all teaching hospitals for 1998/99

**Fig. 10:**
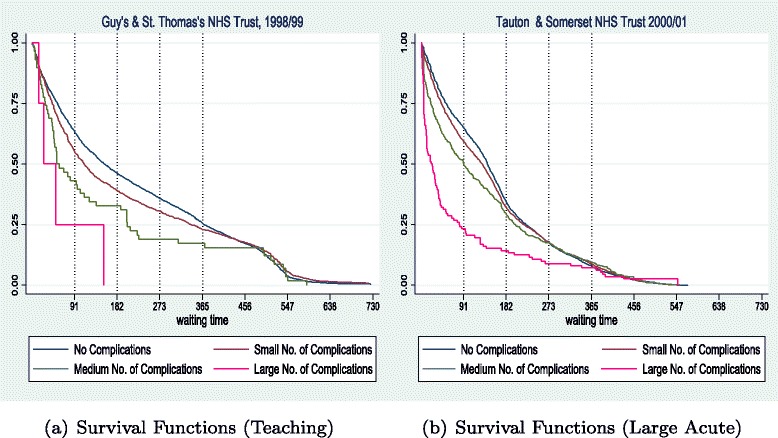
Survival curves by degree of complications for a teaching (**a**) and a large acute (**b**) hospital

Figure 9 presents aggregated results for all teaching hospitals available in our dataset for year 1998/99, comparing the mildest cases (with no complications) and the ones with more than five CC3s. Figure 10 depicts KM survival curves for (a) one of the teaching trusts in the 1998/99 group and (b) for one large acute hospital in 2000/01 (results are robust across hospitals in our dataset).

Our results show that, at the aggregate level, patients with more complications are treated faster throughout the scale of waiting times, particularly at short durations. Hence the largest hump at short waits (in hazard curves) happen for those patients (see Fig. 9(b)). Some more severe cases seem to wait for long, but the number of cases is very small. At the hospital level, while plotting all four categories for degree of severity/complications, we see again a similar pattern. Guy’s & St. Thomas treat cases with more complications faster and with a lower maximum duration, such that no patient from the high group waits beyond 180 days. Whereas, Tauton & Somerset treat the more complex cases faster as well, but maintain a long right tail for the distribution of more severe patients. All in all, there is evidence that hospitals prioritise treatment by clinical severity, particularly at short durations.

### Hospital structural characteristics

As we have seen in the preceding analysis, the empirical waiting time distributions differ across hospitals in both scale, the conditional probability of treatment are higher for some hospitals relative to other for all durations, and shape, indicating that trade-offs between short and long waiters also vary across hospitals. We now use our theoretical model and the empirical survival curves to estimate the main structural parameters that govern the admission patterns of healthcare providers, focusing on the degree of capacity constraint, prioritisation and cost structure to shed some little on those differences.

The empirical exercise employs a minimum distance estimator (MDE) such that the distance between the KM empirical survival function and the one predicted by the theoretical model is minimised. Let *m* *o* *d*_*SF*_(*𝜗*;*ϖ*) be the vector (of length 24) that represents the survival curve^20^ obtained by our model, *𝜗* the subset of 7 parameters, which control the hospital’s admission patterns, to be estimated and *ϖ* the remaining parameters of the model. Finally, let *d* *t* *a*_*SF*_ be the vector that represents the (KM) empirical survival curve estimated in our first empirical exercise. Then the set of parameters estimates $\hat {\vartheta }$ is obtained by

$$\begin{aligned} &\hat{\vartheta} = \arg \min \Omega, \text{where}\; \Omega = \left(dta_{SF} - mod_{SF}(\vartheta; \varpi)\right)\\& \qquad \textbf{W}\left(dta_{SF} - mod_{SF}(\vartheta; \varpi)\right) \text{and } \end{aligned} $$

**W** is a positive definite weighting matrix. For any **W** the (MDE)^21^ is consistent and thus we set **W**=**I**. The vector *𝜗* includes the first four terms of *a*_*d*_,^22^ which determine benefits or prioritisation, *ρ*_0_ and *ρ*_1_, which determine the cost structure and the ratio $Z/\bar {k}$ that determines degree of capacity restriction. While selecting the remaining parameters of the model (*ϖ*), we set the hospital’s budget ($\bar {B}$) and capacity ($\bar {k}$) using the *Hospital Estates and Facilities Statistics* data from the NHS (at the Trust Level), particular the *Estates Service Costs* (in thousands of *$*) for a measure of the hospital’s budget and the *Available beds* as a measure of capacity. Finally, *b*_*d*_, *c*_*d*_ and *θ* are set as in our benchmark model and $\tau = 10\bar {B}/\bar {k}$.

In order to provide some comparison across hospitals we present results for all large acute hospitals (24) in our sample for year 1999/00. The estimated parameters and the list of Large Acute Hospitals and their codes is shown in the Appendix.

**Fig. 11:**
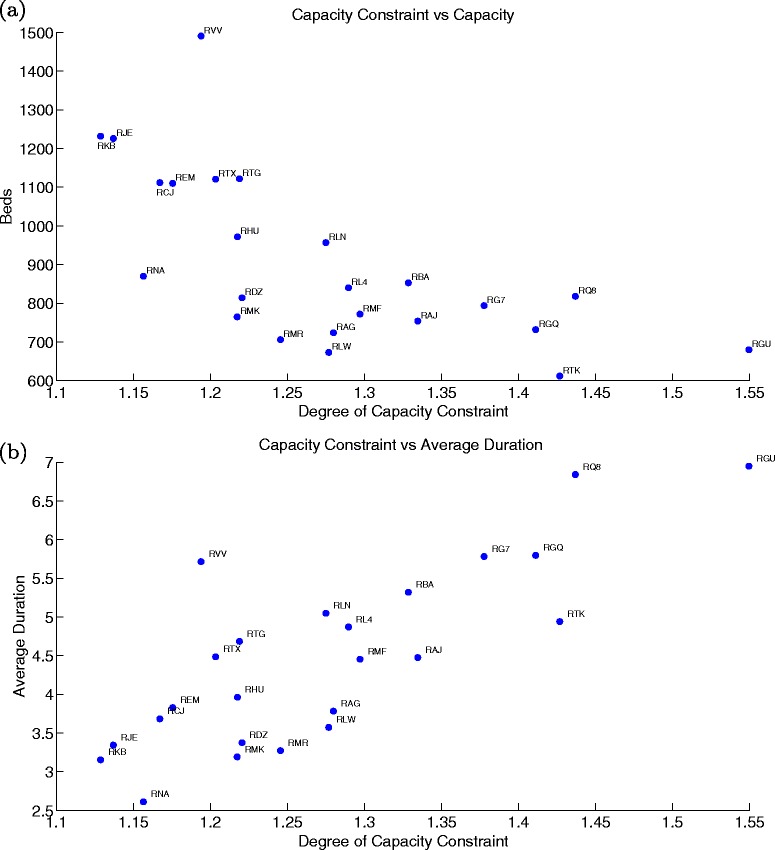
Estimated degree of capacity constraint versus number of beds (**a**) and versus average duration (**b**)

We start by looking at the estimated degree of capacity constraint (ratio of inflow over physical capacity, the latter proxied by the number of available beds). We plot this measure against the number of available beds (Fig. 11(a)) and against the actual average duration of treatment (Fig. 11(b)). As expected greater physical capacity is associated with lower capacity restrictions, which in turn is associated with lower average waiting times. However, some hospitals treat patients slower although they face the same capacity restrictions, and some hospitals although having greater physical capacity are still as restricted as others. How are such variations associated with different degrees of prioritisation and cost structures? Pair-hospital comparisons can be used to highlight the importance of these features.

**Fig. 12:**
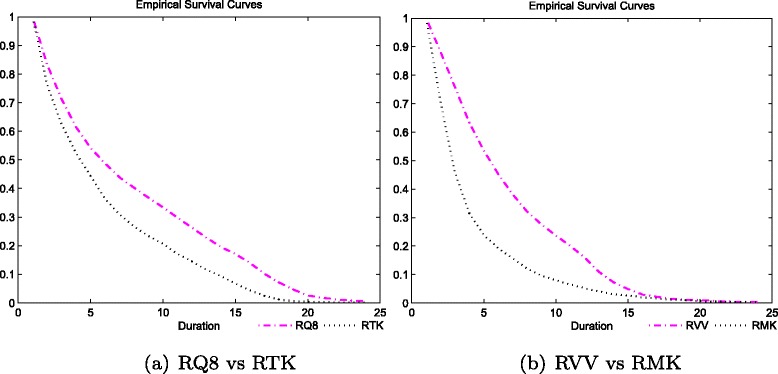
Comparing empirical Survival curves across hospitals: RQ8 vs RTK (**a**) and RVV vs RMK (**b**)

We first compare two hospitals that are relatively more capacity constrained with an inflow around 40 % greater than physical capacity, namely, hospitals RQ8 and RTK. In Fig. 12(a) we plot their empirical survival curves and Table 4 displays the estimated parameters for benefits and costs. Both hospitals face similar degrees of capacity constraints and have similar prioritisation preference. This explains why their survival curves are close to one another for short durations. However, we see a widening of the curves for medium durations. Hospital RTK is able to treat patients of medium duration significantly faster such that its average duration is in fact 2 months lower than the one observed for hospital RQ8. The main reason for the delay in treatment in RQ8 is that the hospital faces a significantly more persistent cost structure (see decay in Table 4). As such, after prioritising patients of short durations, RQ8 is not able to treat medium duration patients fast enough.

**Table 4:** Duration prioritisation and costs

	Benefits				Costs	
	$|\widehat {a}_{1} |$	$|\widehat {a}_{2} |$	$|\widehat {a}_{3} |$	$|\widehat {a}_{4} |$	Level	Decay
RQ8	0.0003	0.0004	0.0005	0.0020	0.3102	0.0015
RTK	0.0002	0.0004	0.0004	0.0019	0.2599	2.1855
RVV	0.0002	0.0004	0.0006	0.0008	0.5168	2.5371
RMK	0.0001	0.0001	0.0005	0.0016	0.3459	3.1598

Further, we compare the characteristics of a small (RVV) and a large (RMK) acute hospital (see Fig. 12(b)). Although both face similar degrees of capacity constraints (20 %), hospital RVV treats patients at a slower pace than RMK, particularly at short durations -the survival curves diverge significantly in the first three periods. This feature is explained by the lack of duration prioritisation (relatively high $|\widehat {a}_{d}|$ for low durations). Hospital RMK has a strong preference to treat as many patients as possible in the first three periods leading to a more efficient management of waiting lists.

**Fig. 13:**
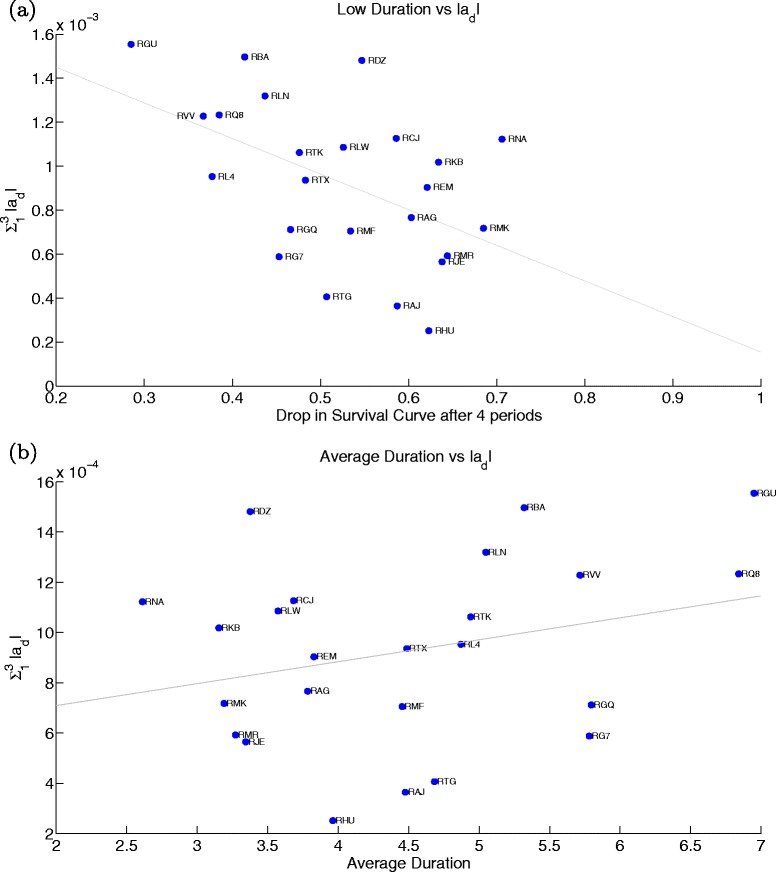
Prioritisation: Estimated benefit structure versus actual drop in survival rates after 4 periods (**a**) and versus average duration (**b**)

**Fig. 14:**
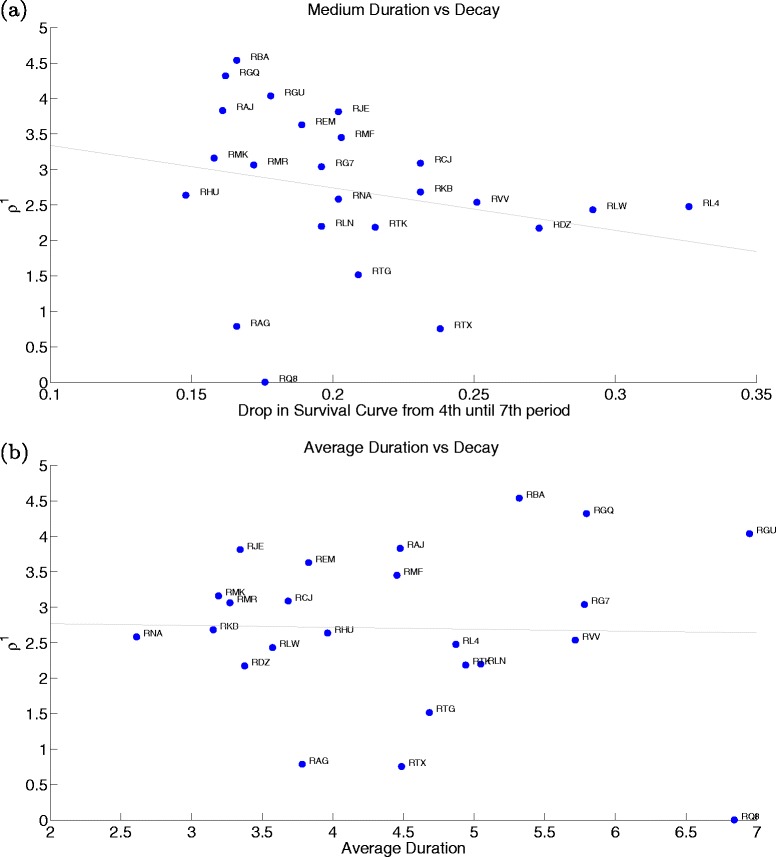
Sensitivity of cost to duration: Estimated cost decay versus actual drop in survival rates from the 4th until 7th period (**a**) and versus average duration (**b**)

In the next two figures we look at all the hospitals included in our estimation and confirm our pair-hospital insights. In the first graph, we plot the estimated ${\sum _{1}^{3}}|\widehat {a}_{d}|$ (benefits structure) versus the actual drop in survival rates for the first four periods of wait (Fig. 13(a)) and versus average duration (Fig. 13(b)). In the second, we see the estimated $\widehat {\rho }^{1}$ (cost decay) against the actual drop in average survival rates from the fourth until the seventh duration (middle portion of the curve) (Fig. 14(b)) and against average duration (Fig. 14(b)). While the benefits structure explains prioritisation of short duration patients and the cost decay explains the treatment profile of medium duration patients well across all hospitals, they are not (or only mildly for ${\sum _{1}^{3}}|\widehat {a}_{d}|$) related to average duration. Therefore, it becomes clear that our identified hospital characteristics provide meaningful information on waiting list management, and most importantly, on the observed trade-offs across patients waiting for treatment.

## Conclusion

We develop a theoretical model of healthcare admission behaviour to study the main drivers of the distribution of waiting times across different healthcare providers. Our theoretical framework has two distinct features: (*i*) the dynamic element of the model and (*ii*) the derivation of the entire optimal waiting time distribution of patients treated at the steady state based on hospital’s structural characteristics.

Using HES data for elective surgery in the UK for years 1997–2005 and duration analysis techniques we also obtain the empirical counterparts of our theoretical waiting time distributions. Looking at survival and hazard functions, we verify vast heterogeneity in the way hospitals are admitting patients for elective surgery. By using the implications of our model and an estimation procedure that compares theoretical and empirical waiting time distributions identifying hospital characteristics, a set of distinct patterns emerge.

On the one hand, some hospitals tend to prioritise duration, ‘front-loading’ treatment and providing healthcare for as many patients as quickly as possible, at the expense however of a fraction that waits for long. Thus, more emphasis is put on short durations. On the other hand some hospitals prefer a more evenly distributed waiting list where patients receive treatment more gradually, but no one waits extensively; the emphasis is on the medium durations. When the treatment specific cost is distinct, we again observe differences in the shape of the survival curves, but now the curvature is altered. When the cost for quick treatment is increased, the survival curves exhibit concave parts (indicating very low treatment rates). Finally, changes in the resources allocated to elective surgery (budget and capacity), relaxing the degree of capacity constraint of a hospital, produce changes in the instantaneous admission rates for the whole distribution, thus we observe shifts in the scale of the survival curves. Further research investigating empirically all supply factors, particularly how the cost structure and hospitals’ objectives change across duration seems to be an important step to understand the rationing of treatment through waiting times, and guide policy design.

## Endnotes

^1^ The terms waiting times and duration will be used interchangeably.

^2^ This reduced form can be obtained by assuming that individuals’ benefits from healthcare decrease while waiting for treatment and that patients have a costly alternative available (e.g. private providers). This is commonly assumed in the literature, with waiting time acting as rationing devise in order to equilibrate demand and supply, similar to what prices do. See for instance [2, 9, 14–19]. Note that extensive expected waiting times can also reduce demand of elective surgeries by discouraging GPs from making referrals.

^3^ Our results do not change if patients have information of a range of possible severities *s* they might suffer from, although the problem becomes considerably more complex since each patient would expect a different expected duration.

^4^ Siciliani [2] makes a similar assumption for average waiting time, while here we focus on the duration of each treatment.

^5^ When $Z<\bar {k}$, the hospital can treat all the patients demanding healthcare will idle capacity ($k_{t}<\bar {k}$), provided that its budget is sufficient. In this case, all patients are treated at *t* and no waiting list is formed.

^6^ This implies it is hard for the hospital to treat patients quickly or equivalently some waiting allows the hospital to reduce costs of providing treatment, using resources more efficiently. Although this negative relationship is well established in the literature, both theoretically ([1]) and empirically ([20]), these contributions also suggest that there might be a level of duration beyond which costs increase (due to higher administrative and medical resources required to manage a long waiting list). We assume that increased costs due to long waits do not occur before *q*.

^7^ The total list of patients of severity *s* at time *t* is given by the current inflow of new patients (*x*_*s*,*t*_) plus all untreated patients from previous periods, $${} \begin{aligned} L_{s, t} = x_{s, t} + \Psi _{2,s,t-1} + \Psi _{3, s, t-1} &+ \Psi _{4, s, t-1}\\ &+ \ldots +\Psi _{q,s,t-1} = x_{s, t} + \sum _{d=2}^{q} \Psi _{d,s,t-1} \end{aligned} $$ and denoting the inflow of patients at *t* as *x*_*s*,*t*_=*Ψ*_1,*s*,*t*−1_, we can write $L_{s,t} = \sum _{d=1}^{q} \Psi _{d,s,t-1}$.

^8^ In the numerical solution the budget value is tied to the treatment cost relative to the hospital’s capacity, representing some sort of a cost-based reimbursement system.

^9^ Although we use a quadratic specification, implying a cost for under utilisation, as long as the budget is ample relative to the treatment-specific cost, conditions we always ensure, optimal $k>\bar {k}$.

^10^ Note that more efficiency might be the result of better management of resources but also lower costs to outsourcing of equipment or personnel.

^11^ We obtain the solution by employing a constrained nonlinear optimisation routine in Matlab. Although it is fairly easy to determine the first and second order conditions of our maximisation problem, these involve many Kuhn-Tucker equations. Thus, it is easier to solve the optimisation problem directly instead of using the resulting system of equations.

^12^ Note that continually increasing physical capacity further might not affect the steady state waiting time distribution. With a given cost structure and budget, the hospital cannot utilise the extra capacity (thus, the budget constraint holds strictly as an inequality). For the list to get shorter, we need to increase the hospital’s budget in line with physical capacity. This result indicates that policies aimed at improving hospital performance as regards waiting lists, must account for both types of investment, namely, monetary budget (flow) and capacity (stock).

^13^ These results are available from the authors upon request.

^14^ Although the budget allocated to elective surgery is exogenous, it is plausible to assume that a different cost and/or capacity structure imply a different budget. In particular, the benchmark budget (B = 7000) has been set proportionally to those two costs (average unit cost (*ρ*_*d*_) times capacity).

^15^ In our context, the ‘event’ of interest is admittance to hospital, ‘survival’ corresponds to remaining on the list, and ‘time’ is that between being placed on a waiting list until admitted for surgery.

^16^ Different selection criteria have been used for presentation of results. Figures 7 and 8 as well as in the Appendix depict hospitals with site codes that remain in the whole sample of 9 years. Figure 10 uses all teaching hospitals for year 1998/99, while the empirical analysis in Section ‘Hospital structural characteristics’ employs all large acute trusts for year 1999/00.

^17^ Log-rank tests, although not reported, confirm significant variation in waiting time distributions in all our cases.

^18^ For more details on the analysis of waiting times, see [6] or [5].

^19^ The hospital payment system in the UK defines a series of diagnostics that are related to complications or more complex cases. This information is used for health resource grouping (HRG) of patients with an aim at measuring extra resource need for each episode. As such, it does provide for a proxy measure of the severity or complexity level of a patient’s case. More information can be found from the Health and Social Care Information Centre (HSCIC): http://www.hscic.gov.uk/article/2322/HRG4-200708-Reference-Costs-Grouper-Documentation

^20^ Waiting times are rarely longer than 2 years.

^21^ See [21] for details.

^22^ We estimate $\widehat {a}_{1}$, $\widehat {a}_{2}$, $\widehat {a}_{3}$ and $\widehat {a}_{4}$, and set $a_{d} = \widehat {a}_{4}+(\widehat {a}_{4}/5 - (\widehat {a}_{4}/5)/(d-4))$ for all *d*>5, such that *a*_*d*_ increases with duration after the forth period. This is done to reduce the number of parameters estimated and since we are concerned with the degree of prioritisation of low duration patients.

^23^At the steady state, $k_{d,s} \leq \Psi _{s,d} \Leftrightarrow k_{d,s} \leq k_{s} - \sum _{h=1}^{d-1}$ $k_{h,s} \Leftrightarrow k_{s} - \sum _{h=1}^{d}k_{h,s} \geq 0 \Leftrightarrow \sum _{h=1}^{q} k_{h,s} - \sum _{h=1}^{d} k_{h,s} \geq 0 \Leftrightarrow \sum _{h=d+1}^{q} k_{h,s} \geq 0$ which holds given that *k*_*d*_,*s*≥0.

## Appendix

### Hospital’s optimisation problem at the steady state

Here we show in more detail the steady state the hospital’s maximisation problem. That is,

$$\begin{aligned} \max_{\{k_{d,s}\}_{d,s}} & \, \, \, \sum_{d=1}^{q} \sum_{s=1}^{p} g(k_{d,s}) \\ \text{Subject to} &\quad \sum_{d} \sum_{s} c(k_{d, s}) + c(k, \bar{k}) \leq B\\ &\quad \, 0 \leq k_{d,s} \leq \Psi_{d,s}\\ & \quad \, k = Z-\theta E(d)\\ & \quad \, \Psi_{d,s} = 0\; \text{for}\; d > q \end{aligned} $$

Recall that $k = \sum _{d} \sum _{s} k_{d,s}$, the steady state expected duration is defined as $E(d) = \sum _{d} d \frac {k_{d}}{k}$ and $\Psi _{d,s} = k_{s} - \sum _{h=1}^{d-1}k_{h,s}$. At the steady state the restrictions that *k*_*d*,*s*_≤*Ψ*_*d*,*s*_ are satisfied as long as *k*_*d*,*s*_ are non-negative.^23^ Thus, the Lagrange function reads:

(3) $${} {\fontsize{8.7pt}{9.6pt}\selectfont{\begin{aligned} \max_{\{k_{d,s}\}_{d,s}} \mathfrak{L} = & \sum_{d} \sum_{s} g(k_{d, s}) + \lambda \left(B - \sum_{d} \sum_{s} c(k_{d, s}) - c(k, \bar{k}) \right) \\ &+ \sum_{d} \sum_{s} v_{d,s}k_{d,s} + + \mu \left(Z-\theta E(d) -k\right) \end{aligned}}}  $$

where *λ* is the lagrangian multiplier of the hospital budget constraint, *v*_*d*,*s*_ is the lagrange multiplier of the Kuhn-Tucker constraint *k*_*d*,*s*_≥0, and *μ* is the multiplier for the condition that ensures that the steady state inflow and outflow are equal.

Solving the hospital’s problem gives rise to 2(*d*×*s*)+2 Karush–Kuhn–Tucker (KKT) conditions. For each *k*_*h*,*m*_ where *h*=1,2,...*q* and *m*=1,2,

$${} {\fontsize{8.4pt}{9.6pt}\selectfont{\begin{aligned} \frac{\partial \mathfrak{L}}{\partial k_{h, m}}& = \frac{\partial \sum_{d} \sum_{s} g(k_{d,s})}{\partial k_{h,m}} - \lambda \left(\!\frac{\partial \sum_{d} \sum_{s} c(k_{d,s})}{\partial k_{h,m}} + \frac{\partial c(k, \bar{k})}{\partial k_{h,m}}\! \right) + v_{h,m} \\ & \quad- \mu \left(\theta \frac{\partial E(d)}{\partial k_{h,m}}+ \frac{\partial \sum_{d} \sum_{s} k}{\partial k_{h,m}} \right) = 0 \\ \frac{\partial \mathfrak{L}}{\partial v_{h,m}} &= k_{h,m} \geq 0, \, v_{h,m} \geq 0 \quad \text{and} \quad v_{h, m} k_{h, m} = 0\\ \frac{\partial \mathfrak{L}}{\partial \lambda} \, & = B - \sum_{d} \sum_{s} c(k_{d,s}) - c(k, \bar{k}) \geq 0, \, \lambda \geq 0 \quad \text{and} \quad \lambda \frac{\partial \mathfrak{L}}{\partial \lambda} =0\\ \frac{\partial \mathfrak{L}}{\partial \mu} & = Z - \theta E(d) - k =0 \end{aligned}}} $$

Given that we do not allow for interaction terms in both the hospital’s utility ($\sum _{d} \sum _{s} g(k_{d,s})$) and the treatment-specific cost ($\sum _{d} \sum _{s} c(k_{d},s)$) functions, the derivative of the Lagrange function with respect to *k*_*h*,*m*_ simplifies to:

$$\begin{aligned} \frac{\partial \mathfrak{L}}{\partial k_{h, m}} &= \frac{\partial g(k_{h,m})}{\partial k_{h,m}} - \lambda \left(\frac{\partial c(k_{h,m})}{\partial k_{h,m}} + \frac{\partial c(k, \bar{k})}{\partial k_{h,m}} \right)\\ &\quad+ v_{h,m} - \mu \left(\theta \frac{\partial E(d)}{\partial k_{h,m}}+ 1 \right)=0. \end{aligned} $$

From this we can derive the optimal number of patients of each severity level treated after having waited *d* durations as a function of all the structural parameters (denoted $\mathfrak {z}$) of the model, $ \, \forall \{d,s\} \quad k^{*}_{d, s} = k^{*}_{d, s} (\mathfrak {z}).$

### Parameter specifications

Tables 5 and 6 show the parameter values and functional forms employed for the benchmark and extended models.

**Table 5:** Benchmark functional specifications and parameters

$g(k_{d})= a_{d}{k_{d}^{3}} + b_{d}{k_{d}^{2}} + c_{d}k_{d}$	Utility from treating *k* patients with duration *d*
where $a_{d} = -0.0002 + \frac {0.0001}{d}$	parameters of the cubic utility function
$b_{d} = 0.02-\frac {0.01}{d}$	
$c_{d} = 2 + \frac {5}{d}$	
*c*(*k*_*d*_)=*ρ*_*d*_ *k*_*d*_	Cost from treatments at duration *d*
where $\rho _{d} = \frac {20}{d^{2}}$	parameter of the linear duration cost function
$c(k)= \tau (k-\overline {k})^{2}$	Scale cost of the total number of patients treated
where $\bar {k}=900$	Hospital’s capacity in terms of number of patients
*τ*=10	sensitivity of cost to deviations from full capacity $\bar {k}$
*B*=7000	Hospital’s budget
*Z*=1200	Potential demand for healthcare
*θ* =50	Sensitivity of inflow to expected waiting time
*q* = 36	Maximum allowed waiting time

**Table 6:** Parameters specification with two levels of severity

$g(k_{d,s})= a_{d,s}{k_{d}^{3}} + b_{d,s}{k_{d}^{2}} + c_{d,s}k_{d}$	Utility from treating *k* patientswith duration *d* & severity *s*
where for the case of low severity:	parameters of the cubic utilityfunction for low severity
*a*_*d*,1_=−0.0002+0.0001/*d*	
*b*_*d*,1_=0.02−0.01/*d*	
*c*_*d*,1_=2+5/*d*	
and for the case of high severity:	parameters of the cubic utilityfunction for high severity
*a*_*d*,2_=0.9(−0.0002+0.0001/*d*)	
*b*_*d*,2_=0.02−0.01/*d*	
*c*_*d*,2_=3+5/*d*	
*c*(*k*_*d*,*s*_)=*ρ*_*d*,*s*_ *k*_*d*,*s*_	Cost from treatments at duration*d* and severity *s*
where *ρ*_*d*,1_=20/*d* ^2^	parameters of the linear duration& severity cost function
and *ρ*_*d*,2_=30/*d*	
$c(k)= \tau (k-\overline {k})^{2}$	Scale cost of the total number ofpatients treated
where $\bar {k}=900$	hospital’s capacity in terms ofnumber of patients
*τ*=10	sensitivity of cost to deviationsfrom full capacity $\bar {k}$
*B*=13500	Hospital’s budget
*Z*=1200	Potential demand for healthcare
*θ* =50	Sensitivity of inflow to expectedwaiting time
*δ*_1_ = 0.7	Proportion of the milderdiagnosis (*s*=1)
*q* = 36	Maximum allowed waiting time

### Empirical analysis - additional results

**Fig. 15:**
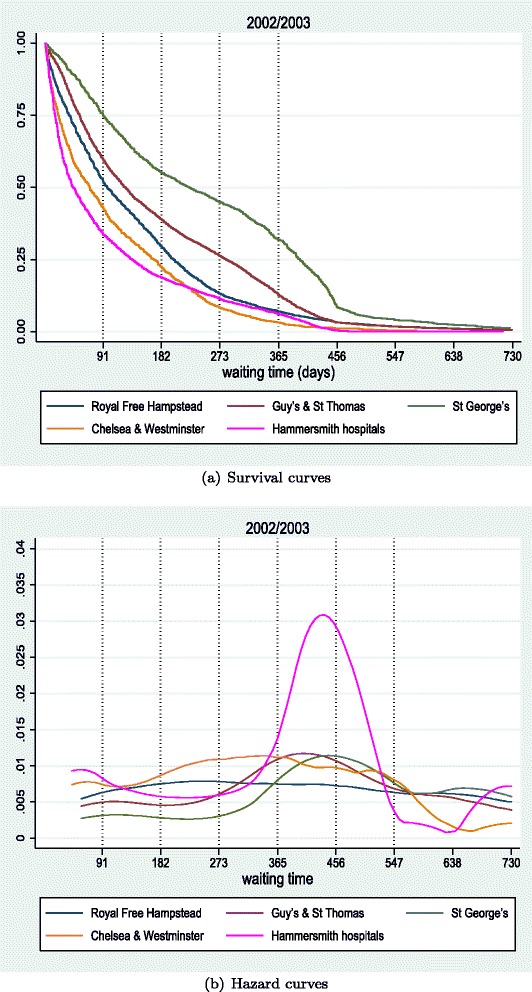
Survival (**a**) and Hazard (**b**) curves for teaching hospitals in London, 2002/2003

**Fig. 16:**
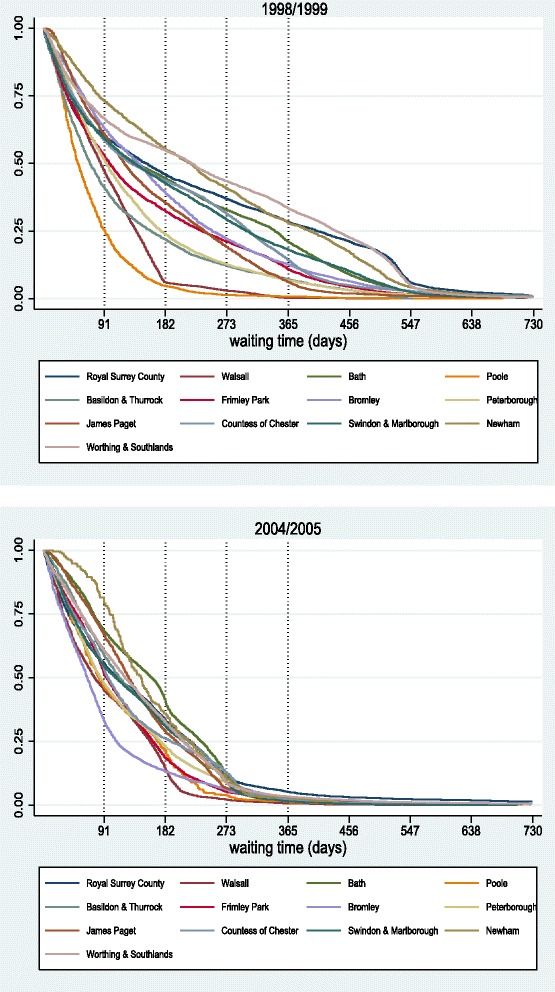
Survival curves for medium acute hospitals, 1998/1999 *(top)* and 2004/2005 *(bottom)*

**Fig. 17:**
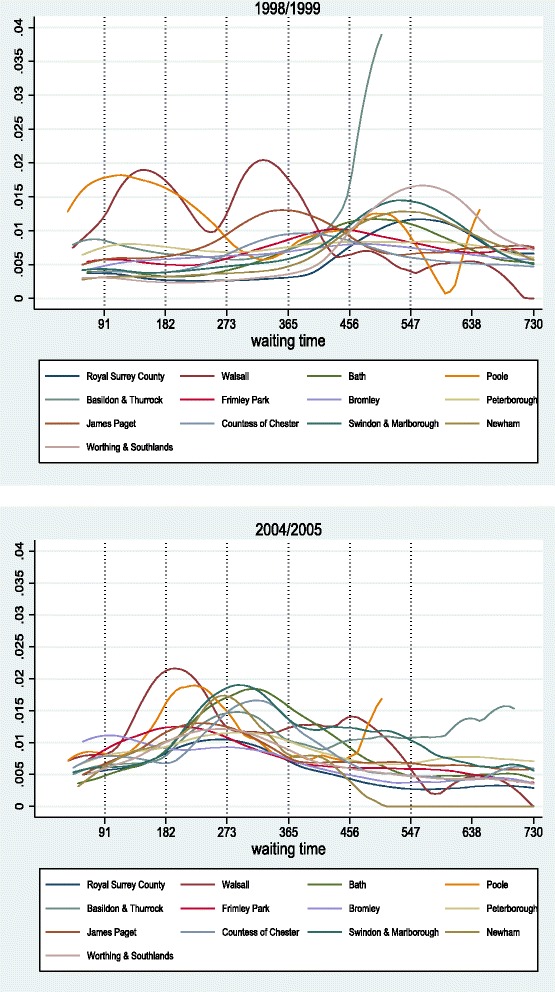
Hazard curves for medium acute hospitals, 1998/1999 *(top)* and 2004/2005 *(bottom)*

**Fig. 18:**
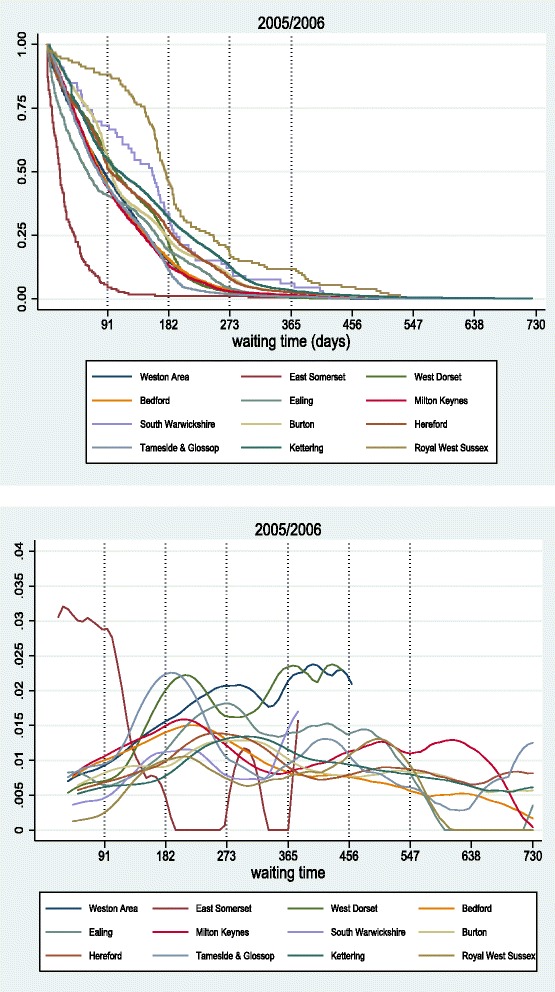
Survival (top) and hazard (bottom) curves for small acute hospitals for 2005/2006

In Fig. 15, 16, 17, and 18, we present additional results of survival and hazard functions for different types of hospitals (teaching, medium acute and small acute). The two key patterns of treatment observed for large acute hospitals are also observed here. Some hospitals concentrate capacity in treating many patients quickly (focus on short waiters), and as a result of scarce resources, are forced to let a sizeable proportion of patients waiting longer until treatment. Other hospitals focus treatment on clearing the list at longer duration, electing not to treat patients quickly but making sure long waits do not occur. Finally, some hospitals are very efficient in treating all patients faster than others.

#### Teaching hospitals

Figure 15 demonstrates the waiting time distributions of a set of seven teaching hospitals in London for years 2002/2003. The admission rates by St George are the worst, and more than 25 % of patients are still awaiting treatment after a year of wait. It is worth mentioning the different tactics by Hammersmith and Chelsea & Westminster hospitals. The former handles quicker the short waiters (< 200 days, where the intersection lies) while delaying admission to long waiters compared with the latter. While looking at the hazard curves, with the exception of Hammersmith that exhibits a high intensity peak between 365 and 456 days, the rest of the hospitals have low intensity wider peaks and Hamstead a constant hazard rate (a result of the smoothest survival curve).

#### Medium acute hospitals

Figures 16 and 17 show the survival and hazard curves for 1998/99 and for 2004/2005 for medium acute hospitals. In the first year of comparison, although hospitals exhibit similar activity levels, they manage quite differently their waiting lists. In 2004/05, all KM curves have shifted leftwards towards the origin and are more concentrated than before. This shows clear response to waiting time targets, as overall waiting times are brought down. Hazard curves confirm this, with observed peaks also moving leftwards. Trade-offs between short and long waiters are still evident. For example, Walsall and Bromley exhibited similar behaviour in 1998/99, but followed different tactics in 2004/05 with the former focusing on long waiters and the latter on handling quickly the short waiters.

#### Small acute hospitals

Figure 18 shows the survival and hazard curves for small acute hospitals for 2005/06. Due to a smaller overall number of admissions, survival curves have more visible steps. We observe a considerable scale difference between East Somerset and Royal West Sussex, with the latter treating short waiters (up to 91 days) quite slowly. The rest of the hospitals are clustered between those two. The hazard curve of East Somerset remains the highest for until about three months, while the one of Royal West Sussex is the lowest. Many trusts exhibit an increased probability of admission at around six months, which is the target of that year.

Tables 7 and 8 depict the list of large acute hospitals and the estimated parameters from the MDE empirical exercise.

**Table 7:** List of large acute hospitals in 1999

Hospital code	Hospital name
RJE	NORTH STAFFORDSHIRE HOSPITAL NHS TRUST
RL4	THE ROYAL WOLVERHAMPTON HOSPITALS NHS TRUST
RLN	CITY HOSPITALS SUNDERLAND NHS TRUST
RTG	SOUTHERN DERBYSHIRE ACUTE HOSPITALS NHS TRUST
RVV	EAST KENT HOSPITALS NHS TRUST
RAG	DONCASTER ROYAL INFIRMARY & MONTAGUE HOSPITAL NHS TRUST
RAJ	SOUTHEND HEALTH CARE NHS TRUST
RBA	TAUNTON & SOMERSET NHS TRUST
RCJ	SOUTH TEES ACUTE HOSPITALS NHS TRUST
RDZ	ROYAL BOURNEMOUTH & CHRISTCHURCH NHS TRUST
REM	AINTREE HOSPITALS NHS TRUST
RG7	HAVERING HOSPITALS NHS TRUST
RGQ	IPSWICH HOSPITAL NHS TRUST
RGU	BRIGHTON HEALTH CARE NHS TRUST
RHU	PORTSMOUTH HOSPITAL NHS TRUST
RKB	WALSGRAVE HOSPITALS NHS TRUST
RLW	THE CITY HOSPITAL NHS TRUST
RMF	PRESTON ACUTE HOSPITALS NHS TRUST
RMK	NORTH MANCHESTER HEALTHCARE NHS TRUST
RMR	BLACKPOOL VICTORIA HOSPITAL NHS TRUST
RQ8	MID ESSEX HOSPITAL SERVICES NHS TRUST
RTK	ASHFORD & ST PETER’S NHS TRUST
RTX	MORECAMBE BAY HOSPITALS NHS TRUST
RNA	THE DUDLEY GROUP OF HOSPITALS NHS TRUST

**Table 8:** Estimated parameters - 24 large acute hospitals 1999

	Benefits				Costs		Capacity constraint
	$|\widehat {a}_{1} |$	$|\widehat {a}_{2} |$	$|\widehat {a}_{3} |$	$|\widehat {a}_{4} |$	$\widehat {\rho }^{0}$	$\widehat {\rho }^{1}$	$\widehat {\tfrac {Z}{\bar {k}}}$
RJE	0.00004	0.00008	0.00044	0.00063	0.2205	3.8135	1.1370
RL4	0.00039	0.00033	0.00024	0.00042	0.6880	2.4762	1.2896
RLN	0.00018	0.00033	0.00081	0.00094	0.4864	2.1974	1.2749
RTG	0.00008	0.00016	0.00017	0.00102	1.6116	1.5153	1.2188
RVV	0.00021	0.00042	0.00060	0.00080	0.5168	2.5371	1.1939
RAG	0.00013	0.00022	0.00043	0.00233	0.0002	0.7880	1.2799
RAJ	0.00004	0.00016	0.00016	0.00069	0.2027	3.8300	1.3346
RBA	0.00018	0.00036	0.00095	0.00075	0.1670	4.5388	1.3285
RCJ	0.00017	0.00034	0.00062	0.00162	0.0000	3.0879	1.1671
RDZ	0.00031	0.00043	0.00074	0.00098	0.0006	2.1725	1.2205
REM	0.00020	0.00007	0.00063	0.00166	0.0002	3.6294	1.1755
RG7	0.00017	0.00021	0.00021	0.00137	0.3819	3.0383	1.3777
RGQ	0.00021	0.00018	0.00032	0.00119	0.3335	4.3211	1.4112
RGU	0.00051	0.00036	0.00068	0.00047	0.1616	4.0379	1.5495
RHU	0.00005	0.00009	0.00011	0.00075	0.7299	2.6364	1.2176
RKB	0.00022	0.00030	0.00050	0.00187	0.0000	2.6826	1.1287
RLW	0.00022	0.00041	0.00046	0.00084	0.5423	2.4319	1.2768
RMF	0.00019	0.00021	0.00031	0.00185	0.4209	3.4504	1.2971
RMK	0.00010	0.00009	0.00052	0.00158	0.3459	3.1598	1.2173
RMR	0.00005	0.00017	0.00037	0.00075	0.3988	3.0638	1.2455
RQ8	0.00031	0.00043	0.00050	0.00197	0.3102	0.0015	1.4369
RTK	0.00020	0.00045	0.00041	0.00194	0.2599	2.1855	1.4268
RTX	0.00025	0.00034	0.00035	0.00157	0.3587	0.7540	1.2034
RNA	0.00024	0.00026	0.00062	0.00175	0.0000	2.5812	1.1564
